# Study on the Wear and Corrosion Resistance of PEO/SAM/MWCNTs Composite Coating on TC4/Mg Interpenetrating Composite

**DOI:** 10.3390/ma19112292

**Published:** 2026-05-28

**Authors:** Xinyan Dong, Ben Ma, Jianwei Hu, Qing Wu, Yunlong Zhang, Chenghai Li, Tao Jiang, Hehe Chen, Long You

**Affiliations:** 1 AVIC Touchstone Testing Innovation (Wuxi) Co., Ltd., Wuxi 214101, China; dxy15545763716@163.com (X.D.);; 2 College of Materials Science and Engineering, Jiamusi University, Jiamusi 154007, China; 3 AECC Beijing Institute of Aeronautical Materials, Beijing 100095, China

**Keywords:** TC4/Mg interpenetrating phase composite, plasma electrolytic oxidation (PEO), self-assembled monolayer, multi-walled carbon nanotubes, friction and wear, corrosion protection

## Abstract

**Highlights:**

- PEO coating on TC4/Mg composite consists of Mg_2_SiO_4_, MgO, MgF_2_ and TiO_2_.
- PEO + SAM coating with MWCNTs fully seals micropores, forming dense layered surface.
- Composite coating reduces wear volume by 93.53% vs. substrate, COF drops to 0.195.
- Corrosion current density decreases from 2 × 10^−4^ to 1.401 × 10^−9^ A·cm^−2^ (5 orders).
- MWCNTs act as nano-rolling bearings, reducing friction and adsorbing wear debris.
- Multi-level “ceramic barrier + nano-sealing + molecular protection” system is built.
- The composite coating offers a new solution for wear/corrosion protection of implants.
- Strategy may be extended to other interpenetrating phase composite materials.

**Abstract:**

To address the severe wear and galvanic corrosion of TC4/Mg three-dimensional interpenetrating composites caused by the potential difference and hardness disparity between the two phases, this work proposes a hybrid surface modification strategy combining plasma electrolytic oxidation (PEO) with a self-assembled monolayer (SAM) doped with multi-walled carbon nanotubes (MWCNTs). A PEO ceramic coating was first grown in situ on the composite surface, followed by sealing modification using MWCNTs-containing SAM. The microstructure, phase composition, tribological behavior and potentiodynamic polarization curves of the coatings were systematically evaluated. The results show that the PEO coating is mainly composed of Mg_2_SiO_4_, MgO, MgF_2_ and TiO_2_, exhibiting a typical porous structure. After the MWCNTs-doped SAM composite modification, the nano-fillers and the molecular layer synergistically seal the micropores and cracks, and the surface transforms into a continuous and dense layered morphology. Wear tests reveal that the composite coating reduces the friction coefficient to 0.195 and decreases the wear volume by 93.53% compared with the bare composite. The “micro-roller bearing” effect and debris adsorption of MWCNTs significantly improve the wear resistance, and the dominant wear mechanism changes from abrasive wear to three-body wear. Electrochemical measurements show that the corrosion current density of the composite coating decreases from 2 × 10^−4^ A·cm^−2^ (bare composite) to 1.401 × 10^−9^ A·cm^−2^, i.e., a reduction by five orders of magnitude, with a protection efficiency of 99.99%. This is attributed to the physical barrier effect of the PEO coating and the synergistic sealing of defects, as well as the blocking of electron transfer by MWCNTs/SAM. The multi-level protection system of “PEO + MWCNTs + SAM” constructed in this work achieves a synergistic improvement in both wear resistance and corrosion resistance of the TC4/Mg two-phase interpenetrating composite, and holds promise for further investigation as an osseointegration implant material.

## 1. Introduction

With the growing demand for lightweight high-strength materials in aerospace, marine engineering, and biomedical applications, metal–metal interpenetrating phase composites (MMIPCs) have emerged as a research hotspot in the field of lightweight structural materials due to their high specific strength, topologically continuous architecture, and multi-scale interpenetrating characteristics [1]. Titanium alloys exhibit high specific strength, excellent corrosion resistance, and good biocompatibility, making them ideal materials for critical aerospace components and medical implants [2,3]. Magnesium alloy has a density of only about 1.74 g/cm^3^, and its specific strength is much higher than that of aluminum alloys and steel, offering significant advantages in weight reduction and energy saving [4,5]. However, the inherent limitations of titanium alloys (e.g., high density of ~4.43 g/cm^3^) and magnesium alloys (e.g., poor corrosion resistance and low hardness) restrict their individual application in harsh service environments [6,7,8]. Three-dimensional interpenetrating phase composites, through the design of a continuous three-dimensional network skeleton and an interpenetrating filler phase, can achieve a synergistic “strong–tough–light” effect. In recent years, TC4 (Ti-6Al-4V)/Mg interpenetrating composites have attracted considerable attention owing to their excellent mechanical properties [9,10,11,12]. Although the introduction of TC4 improves the mechanical performance and corrosion behavior of the magnesium matrix to some extent [13,14], the significant galvanic potential difference (approximately 1.5 V) between TC4 and Mg induces galvanic corrosion, while poor wear resistance remains a critical bottleneck restricting the engineering application of this composite.

To address the issues introduced by dissimilar metal coupling structures, plasma electrolytic oxidation (PEO) has emerged as a highly promising surface treatment technique because it can in situ grow ceramic coatings on valve metals, thereby improving the low hardness, poor wear resistance, and inadequate corrosion resistance of the substrate [15,16,17,18,19]. However, the inherent porous structure and micro-cracks of PEO coatings still constitute weak points for long-term protective performance [20]. To remedy these structural defects of PEO coatings, post-treatment sealing technologies have developed rapidly in recent years.

Self-assembled monolayers (SAMs) exhibit broad application prospects in surface modification due to their advantages of spontaneous in situ formation, highly ordered bonding arrangement, few defects, and strong adhesion. Through chemical bonding of long-chain silane or phosphonic acid molecules onto the PEO coating surface, SAMs can fill micropores and micro-cracks, forming a dense hydrophobic barrier [21]. Meanwhile, carbon nanotubes (CNTs), with their unique three-dimensional tubular structure and excellent self-lubricating properties, have been widely used to enhance the mechanical and tribological performance of composite coatings [22,23,24]. Li et al. [25] reported that the synergistic effect of CNTs and the porous structure of PEO reduced the friction coefficient by approximately 43.14%. Li et al. [26] prepared a PEO/polyimide (PI) composite coating on AZ91 magnesium alloy by applying a PI top layer onto the PEO coating; the sealing and filling of surface micropores by the PI layer reduced the corrosion current density by three orders of magnitude, while the strong adhesion and lubrication of PI endowed the composite coating with self-lubricating properties. These studies indicate that combining PEO coatings with post-treatment sealing and nano-reinforcement is an effective route to achieve synergistic wear and corrosion protection.

Nevertheless, current research has the following limitations: (1) The formation mechanism of PEO coatings on the heterogeneous and interpenetrating dual-complex structure of TC4/Mg interpenetrating composites has not been deeply explored. (2) The synergistic application of SAM self-assembled sealing and MWCNT nano-reinforcement for post-treatment of PEO coatings has not yet been reported; in particular, the dispersion of functionalized MWCNTs in SAM solutions and their cooperative assembly mechanism on the PEO coating surface remain unexplored.

To address the above issues, this work designs and fabricates TC4/Mg three-dimensional interpenetrating composites and their surface PEO coating, as well as PEO/SAM/MWCNTs composite coatings. The microstructure, wear resistance, and corrosion resistance of each coating system are systematically investigated. By comparatively analyzing the stepwise performance enhancement from the TC4/Mg composite to the PEO/SAM/MWCNTs composite coating, this study elucidates the blocking mechanism of the PEO coating against galvanic corrosion at the TC4/Mg heterogeneous interface, reveals the synergistic enhancement effect of SAM sealing and MWCNT nano-reinforcement on the wear and corrosion resistance of the coatings, and provides a theoretical basis and technical reference for the surface protection of TC4/Mg three-dimensional interpenetrating composites under harsh environments.

## 2. Materials and Methods

### 2.1. Experimental Materials

**Figure 1:**
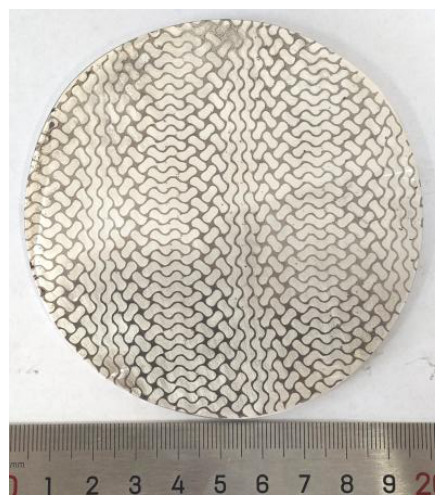
Macroscopic photo of TC4/Mg three-dimensional interpenetrating composite material.

The substrate material was TC4/Mg three-dimensional interpenetrating composite fabricated by the pressure infiltration method. The TC4 titanium alloy scaffold had a porosity of approximately 72%, and the magnesium alloy (AZ31) was infiltrated into the TC4 scaffold pores by vacuum pressure infiltration, forming a bicontinuous three-dimensional interpenetrating structure. The macroscopic morphology is shown in Figure 1. The composite was cut into square specimens with dimensions of 20 mm × 20 mm × 2 mm by wire electrical discharge machining. Prior to the experiment, the specimens were successively ground with 120#, 400#, 800#, 1200#, and 2000# metallographic abrasive papers, then ultrasonically cleaned with acetone for 10 min, rinsed with deionized water, and dried with cold air. The substrate was designated as S0.

### 2.2. Experimental Methods

#### 2.2.1. Preparation of PEO Coating

The ceramic coating was prepared on the substrate surface using a bipolar pulse power supply. The base electrolyte composition for plasma electrolytic oxidation (PEO) was: 20 g/L Na_2_SiO_3_, 3 g/L EDTA-2Na, 10 g/L NaF, 5 g/L NaOH, and 6 mL/L glycerol. The PEO electrical parameters were: voltage 360 V, treatment time 8 min, frequency 450 Hz, and duty cycle 0.2. During the process, circulating water was used to cool the electrolyte, maintaining the electrolyte temperature at approximately 25 °C. After the experiment, the specimens were rinsed with distilled water, dried, and designated as S1.

#### 2.2.2. Preparation of SAM Composite Coating

The TC4/Mg-PEO specimens were pretreated by immersion in a 3 mol/L sodium hydroxide solution in a constant-temperature water bath at 60 °C for 1 min, followed by drying. The self-assembling solution consisted of: 5% silane solution (with a volume ratio of ethanol:water:silane (DPTMS) = 85.5:9.5:5), 2% phytic acid solution, and MWCNTs. The prepared 5% silane solution was allowed to stand at room temperature for 12 h, after which 3 g/L MWCNTs were added and stirred evenly for the experiment. The TC4/Mg-PEO specimens were separately immersed in the phytic acid and silane solutions for 30 s each, then dried at 60 °C for 8 min. This immersion and drying cycle was repeated six times. The resulting specimens were designated as S2. During the experiment, positive and negative electrolytes were placed on a magnetic stirrer to maintain solution uniformity.

#### 2.2.3. Friction and Wear Test

A pin-on-disk tribometer (SFT-2M) was used to evaluate the wear performance of S0, S1, and S2. The counterface material was a ZrO_2_ ceramic ball (Φ6 mm) with a hardness slightly higher than that of the PEO coating (approximately 1200 HV1), which exhibits good biocompatibility and excellent wear resistance [27]. To avoid premature coating failure and to enable an accelerated, effective, and realistic assessment of its wear resistance [28], the applied load was set to 0.6 N, the wear track radius to 3.2 mm, the rotational speed to 200 r/min, and the test duration to 20 min.

#### 2.2.4. Electrochemical Corrosion Test

Potentiodynamic polarization curves of S0, S1, and S2 were measured using an electrochemical analyzer (VersaSTAT3). The test solution was 3.5 wt.% NaCl solution. A standard three-electrode system was employed: an Ag/AgCl electrode as the reference electrode, a platinum electrode as the counter electrode, and the specimen as the working electrode with a testing area of 1 cm^2^. The scan rate was 1 mV/s, with an initial potential of −0.5 V and a final potential of 0.75 V. The measured voltage–current data were plotted as Tafel curves in E-log i format.

### 2.3. Analysis and Characterization

A Bruker D8 X-ray diffractometer (Bruker AXS GmbH, Karlsruhe, Germany) was used for phase analysis of the specimens, with a scanning range of 20–90° and a scanning speed of 6°·min^−1^. A TESCAN-S8000 (TESCAN ORSAY HOLDING, Brno, Czech Republic) field emission scanning electron microscope (SEM) equipped with an Aztec energy dispersive spectrometer (EDS) was employed to observe the surface and cross-sectional morphologies of the coatings as well as the worn surface morphologies, and to perform elemental analysis. ImageJ software (ImageJ 1.54f) was used to quantitatively analyze the surface porosity of the PEO coatings. A microhardness tester (Qness Q10A^+^, Qness GmbH, Golling an der Salzburg, Austria) was used to measure the Vickers hardness of the coating surfaces under a load of 0.2 N with a dwell time of 12 s. Five measurements were taken on each sample, and the average value was taken with a controlled error of within 10%. An Olympus laser confocal measuring microscope (LEXT-OLS 4000, Olympus Corporation, Tokyo, Japan) was used to analyze the three-dimensional morphology of the wear scars and to characterize the wear volume, wear depth, and profile curves.

To ensure data reliability, all quantitative tests were repeated at least three times (*n* ≥ 3), and the results are presented as mean ± standard deviation (SD). Coating thicknesses were measured at five randomly selected positions on the TC4 side and on the Mg side, and the average value was taken. The wear volume was obtained by integrating the wear scar profile using the laser confocal microscope, and each specimen was measured three times. The friction coefficient was taken as the average value over the steady-state stage (200–1200 s), and each test was repeated three times; the error bars were calculated from the standard deviation of the three repeated experiments. All data were statistically analyzed using Origin 2021 software.

### 2.4. Declaration of Generative AI and AI-Assisted Technologies in the Writing Process

In the preparation of this manuscript, the authors used AI language models solely for optimizing the language expression and grammar of certain paragraphs. All AI-generated content was incorporated into the final manuscript under the authors’ manual review and revision. The authors take full responsibility for all content of the manuscript, including the parts where AI tools were used. AI tools were not listed as authors.

## 3. Results and Discussion

### 3.1. Phase Composition Analysis

**Figure 2:**
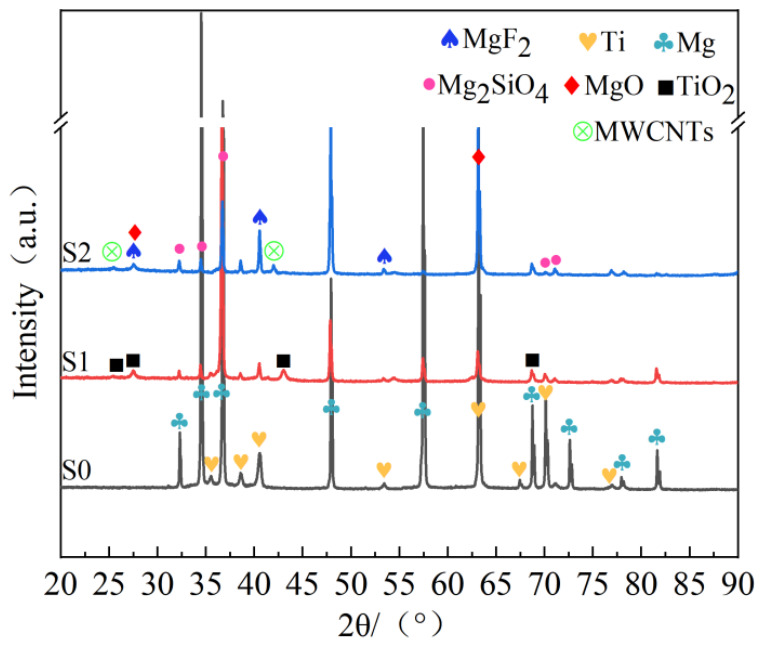
XRD spectrum.

The phase compositions of the TC4/Mg three-dimensional interpenetrating composite and its surface coatings were analyzed by X-ray diffraction (XRD), and the results are shown in Figure 2. From the XRD patterns, it can be seen that the TC4/Mg interpenetrating composite only exhibits diffraction peaks of Ti and Mg, with no characteristic peaks of other phases detected. After PEO treatment, the coating is mainly composed of Mg_2_SiO_4_ (Forsterite, ICDD PDF #85-1364), MgO (Periclase, ICDD PDF #45-0946), MgF_2_ (Sellaite, ICDD PDF #38-0882), TiO_2_ (Anatase, ICDD PDF #21-1272), and the substrate phases (Ti and Mg). The formation of the Mg_2_SiO_4_ phase originates from the participation of silicate components in the electrolyte during the PEO discharge reaction, while the appearance of MgF_2_ is attributed to the fluorine-containing electrolyte. It is worth noting that the XRD patterns still show strong substrate diffraction peaks. This is because the PEO coating is relatively thin and porous, allowing X-rays to easily penetrate through the coating pores to reach the substrate, thereby generating strong substrate signals. After SAM composite modification on the basis of the PEO coating, the main crystalline phases of the composite coating do not change significantly. However, new diffraction peaks are observed at approximately 25.44° and 42.09° in the XRD pattern, which are assigned to the (002) and (100) planes of graphitic MWCNTs, respectively (ICDD PDF #00-056-0159). Although the additional amount of MWCNTs is low, their characteristic diffraction peaks are still clearly identifiable, indicating that MWCNTs have been successfully incorporated and embedded into the PEO-SAM composite coating system. This result confirms the effective introduction of MWCNTs during the composite modification process, providing a structural basis for further improving the density and protective performance of the coating.

### 3.2. Microstructure Analysis

**Figure 3:**
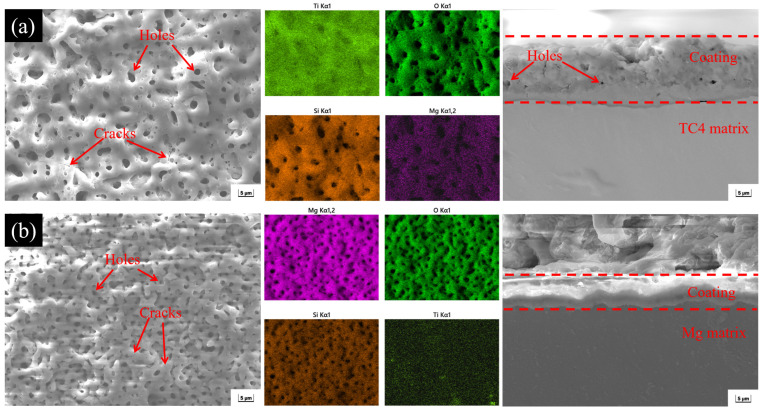
SEM images of the coating surface and cross-section of the S1 specimen, along with the corresponding EDS elemental distribution maps of surface Ti, Si, O, and Mg; (**a**) TC4-PEO; (**b**) Mg-PEO.

Figure 3 shows the surface and cross-sectional morphologies of S1, along with the surface elemental distribution. A PEO coating was successfully prepared on both the TC4 titanium alloy and the magnesium (Mg) matrix. The microstructures on the two phases are consistent, both exhibiting approximately circular micropores and “sintered disc”-like molten solidified structures. The formation of this porous structure is closely related to the escape of gas bubbles from the discharge channels and the jetting flow of molten oxides. During the PEO process, intense spark discharges cause the local surface temperature to instantaneously reach 2000–10,000 K, leading to dielectric breakdown and molten flow, thereby forming pores in situ. The molten material rapidly cools in the electrolyte and adheres to the substrate surface, ultimately forming micropores and “sintered disc” structures. In addition, a few microcracks exist on the coating surface, which are attributed to thermal mismatch stress caused by the rapid solidification of high-temperature molten oxides in the relatively cold electrolyte.

**Figure 4:**
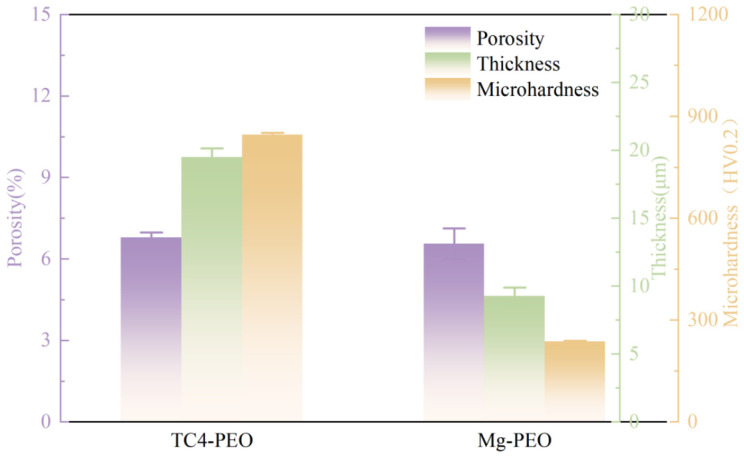
Surface porosity, thickness, and microhardness of the two-phase PEO coatings.

The surface porosity, thickness, and microhardness of the two-phase PEO coatings are shown in Figure 4. On the TC4 side, the coating surface exhibits larger “sintered discs” and larger pore sizes, with a porosity of 6.78 ± 0.19%. On the Mg side, the “sintered discs” are smaller, the pore sizes are smaller, and there are more pores, with a porosity of 6.55 ± 0.57%. Elemental analysis reveals that the main elements in the coatings on both the TC4 and Mg sides are Ti, Si, O, and Mg, with no other impurity elements introduced. Ti originates from the TC4 substrate, Mg from the Mg alloy matrix, Si from the Na_2_SiO_3_ electrolyte, and O from the oxidation reaction during PEO treatment. The coating thickness on the TC4 side is 19.49 ± 0.65 μm, and its microhardness reaches 845.8 ± 5.6 HV0.2. Its internal structure consists of a loose layer, a transition layer, and a dense layer. The loose layer has a relatively rough surface and contains many closed micropores. The dense layer is located at the interface between the substrate and the coating; it is the most compact layer and is free of micropores. The coating thickness on the Mg side is 9.25 ± 0.63 μm, and its microhardness reaches 235.2 ± 3.3 HV0.2. Its internal structure is relatively dense, with almost no defects observed.

The significant differences in surface morphology and cross-sectional structure of the PEO coatings between the TC4 side and the Mg side arise from the coupled effects of their intrinsic physicochemical properties and discharge behaviors. On the TC4 side, owing to its high melting point, low thermal conductivity, and high dielectric constant of TiO_2_ [29,30,31], the single discharge energy is large and the molten material flows abundantly, resulting in large “sintered discs” and large pores. Meanwhile, due to the volume expansion effect and the deep heat-affected zone [32,33], the coating develops into a three-layer structure of a loose layer, a transition layer, and a dense layer, reaching a thickness of 19.49 μm. On the Mg side, because of its low melting point, high thermal conductivity, and low dielectric constant of MgO [34,35], the discharge sparks are small and dense, and the molten material solidifies rapidly, forming small “sintered discs” and fine pores. Furthermore, volume shrinkage and a shallow heat-affected zone lead to a single, uniform, and dense coating structure with a thickness of only 9.25 μm. The similar porosity values on both sides indicate comparable coating-formation efficiency, whereas the differences in microstructure and layered architecture are essentially a natural consequence of the distinct thermophysical and dielectric characteristics of the two materials.

**Figure 5:**
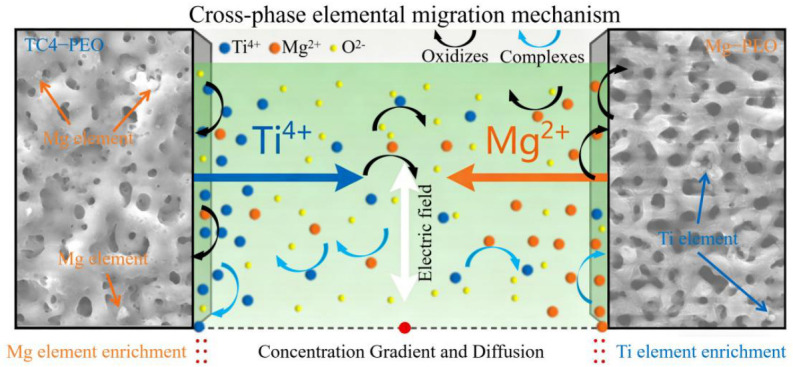
Schematic diagram of interphase material transfer mechanism.

Notably, during the PEO process, Mg enrichment appears in the coating on the TC4 side, while Ti enrichment appears in the coating on the Mg side. This cross-phase migration phenomenon may be attributed to the synergistic effect of three mechanisms: plasma sputtering and re-deposition, electric-field-driven migration, and concentration-gradient diffusion. A schematic diagram of the cross-phase material transfer mechanism is shown in Figure 5. During PEO, the high-temperature plasma generated by micro-arc discharges causes melting and vaporization of material near the discharge channels, which are then sputtered outward as nano-sized particles. These high-energy species (Ti^4+^, Mg^2+^, and their oxides) are ejected from one surface into the electrolyte and onto the opposite surface. Since both TC4 and Mg act as anodes, the Ti^4+^ and Mg^2+^ ions ionized under high voltage form negatively charged complexes with EDTA-2Na [36,37]. Under the electric field, these complexes migrate and re-adsorb onto the surfaces of both phases. Moreover, the concentration gradients of Ti^4+^, Mg^2+^, and their oxides near the two-phase surfaces further enhance the cross-phase material exchange.

**Figure 6:**
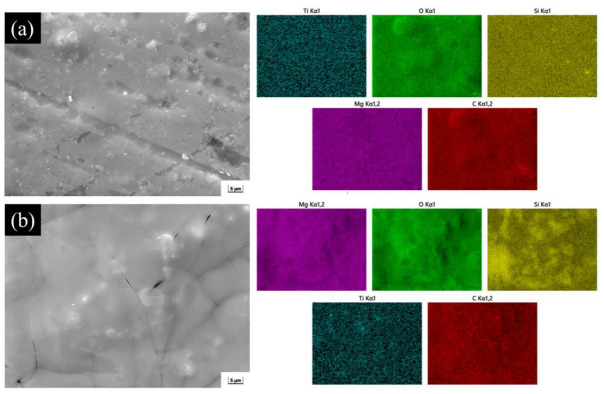
SEM images of the coating surface of S2 samples and corresponding EDS elemental distribution maps of Ti, Si, O, and Mg; (**a**) TC4-PEO; (**b**) Mg-PEO.

After modification with MWCNTs doping and SAM composite treatment, the original micron-sized pores and defects of the two-phase PEO coating are completely sealed by the nano-fillers and the self-assembled molecular layer. The surface transforms from an open porous structure into a continuous, flat, and uniform layered morphology. This indicates that the nano-filling effect of MWCNTs and the molecular-level coverage of SAM synergistically achieve precise repair of the coating defects, as illustrated in Figure 6.

### 3.3. Analysis of Coating Friction and Wear Performance

**Figure 7:**
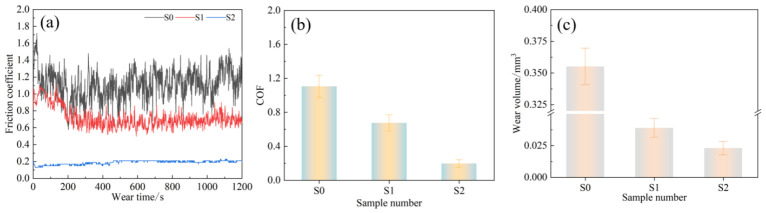
Wear resistance test results: (**a**) friction coefficient curve; (**b**) average friction coefficient; (**c**) wear volume.

In this study, a ball-on-disk wear test (duration 20 min) was conducted to evaluate the wear resistance of the substrate and coatings by analyzing the friction coefficient (COF), wear ring width, wear track depth, and wear volume. Figure 7 presents the wear test results of the TC4/Mg three-dimensional interpenetrating composite and its coatings. From Figure 7a, it can be seen that during the initial 200 s of the test, the COF gradually increases and then levels off. This evolution is closely related to the initial surface morphology of the material. Throughout the whole friction process, the COF of the bare composite fluctuates considerably, ranging from 0.434 to 0.526. This phenomenon can be attributed to the hardness difference between the TC4 phase and the Mg phase: the friction behaviour at the interface between the hard TC4 phase and the soft Mg phase differs from that inside each individual phase. When the contact point switches between the phase interface and the interior of a phase, the COF exhibits significant fluctuations. After PEO treatment, the fluctuation amplitude of the COF curve of the coated surface is markedly reduced, although small-scale fluctuations remain. These residual fluctuations mainly originate from differences in hardness and roughness between the coating on the TC4 side and that on the Mg side, causing changes in friction behaviour when the sliding pair moves from one coated phase to the other. After SAM/MWCNTs composite modification, the COF curve of the coated surface becomes essentially stable. From Figure 7b, the average COF values of the bare composite, the PEO-coated sample, and the SAM composite-coated sample are 1.105 ± 0.130, 0.674 ± 0.095, and 0.195 ± 0.045, respectively. It is evident that the coatings can effectively reduce the friction coefficient, with a maximum reduction of 82.35%. The MWCNTs in the SAM composite coating possess a unique nanotubular structure, forming a “micro-roller bearing” effect inside the coating. During relative motion, these nanotubes can roll between the coating and the counterface, reducing direct sliding friction and thereby significantly lowering the COF. At the same time, MWCNTs have a strong physical adsorption capacity, which allows them to adsorb wear debris and other impurities generated during friction, preventing debris accumulation at the contact interface that would otherwise aggravate friction. Consequently, the friction process becomes smoother and the COF more stable.

**Figure 8:**
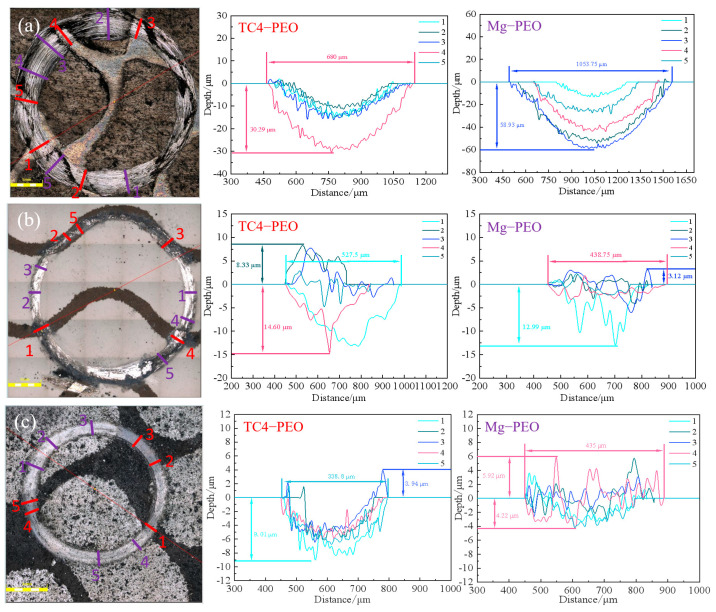
Macroscopic morphology of the surface wear areas and wear profile curves: (**a**) S0; (**b**) S1; (**c**) S2.

Figure 8 shows the two-dimensional morphology of the worn surfaces and the wear profile curves of the two-phase regions (the measured areas include slight, moderate, and severe wear zones). Comparing the wear morphology and profile curves (Figure 8a), the worn area of the bare composite exhibits a metallic luster, and the wear degree of the two phases differs markedly. On the TC4 phase, the maximum wear scar width is 680 μm and the maximum wear depth is 30.29 μm; on the Mg phase, the maximum wear scar width is 1053.75 μm and the maximum wear depth is 58.93 μm. The wear volume is 0.3551 mm^3^. From Figure 8b, after PEO treatment, the worn surface of the sample shows both white and black regions: the black region corresponds to the coating on the TC4 side, while the white region corresponds to the coating on the Mg side. This colour difference may be attributed to the fact that, during wear, the local high temperature and friction cause a change in the oxidation state of titanium, resulting in the formation of black or grey titanium oxide compounds (e.g., Ti_2_O_3_) [38]. In contrast, under wear conditions, magnesium mainly forms stable white magnesium oxide, which has a relatively uniform colour, so the coating on the Mg side remains white. In the severe wear zone, the wear depths of the coatings on the TC4 side and the Mg side are 14.60 μm and 12.99 μm, respectively, and the maximum wear scar widths are 527.5 μm and 438.75 μm, respectively. The wear volume is 0.03889 mm^3^. From Figure 8c, after SAM/MWCNTs composite modification, the wear traces on both sides become more uniform: the maximum wear depths of the composite coatings on the TC4 side and the Mg side are 9.01 μm and 4.22 μm, respectively, and the maximum wear scar widths are 338.8 μm and 435 μm, respectively. The wear volume is 0.02298 mm^3^. Thus, the coating significantly reduces the wear depth and width of the substrate, with a maximum reduction in wear volume of 93.53%.

**Figure 9:**
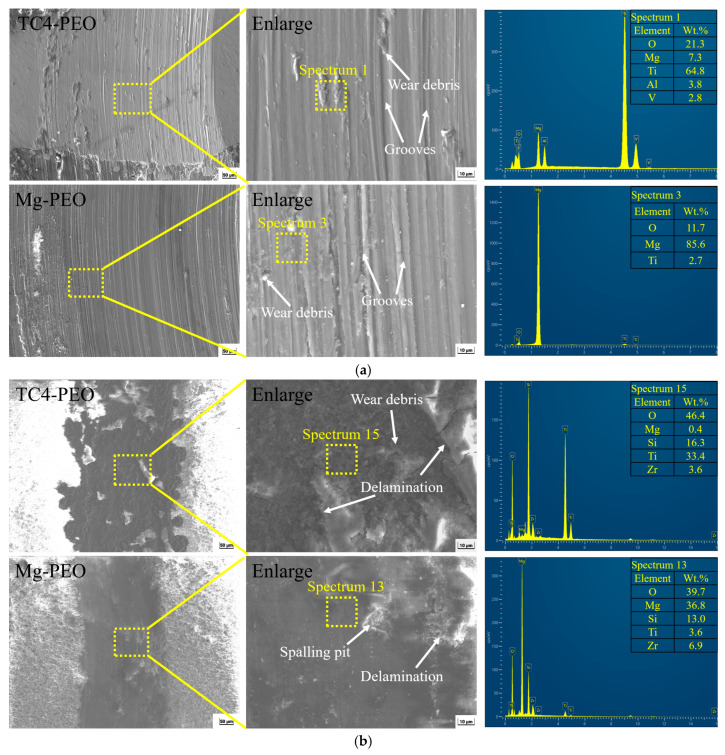

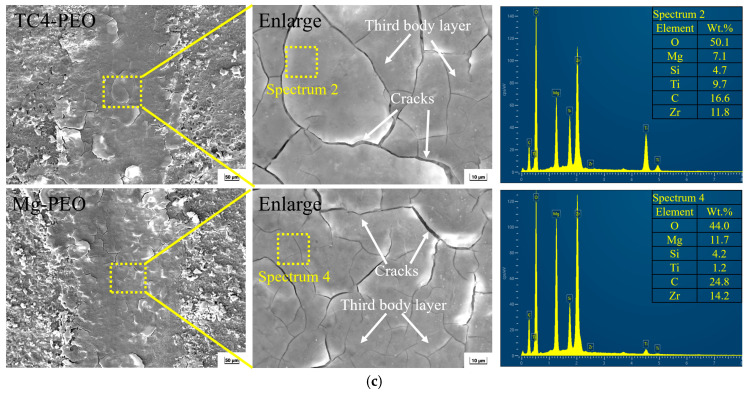
SEM morphology and EDS spectra of the worn surface: (**a**) S0; (**b**) S1; (**c**) S2.

To further analyze the wear mechanism, the surface morphologies of the two-phase worn areas were observed at the microscopic level (Figure 9). From Figure 9a, it can be seen that in the two-phase worn areas of the bare composite, both wear debris accumulation and obvious grooves are present, and the magnified images reveal fine granular wear debris. This originates from the debris generated between the composite and the ZrO_2_ counterface during friction, which produces a plowing effect on the coating surface under tangential stress. Therefore, the dominant wear mechanism under the present test conditions is abrasive wear, accompanied by oxidative wear. From Figure 9b, after PEO treatment, numerous adhered substances and plastic deformation can be observed on the worn surface. The typical porous structure of the PEO coating is beneficial for storing wear debris. Under the synergistic action of cyclic contact stress and frictional heat, the debris is compacted and oxidized, gradually forming a third-body layer that protects the ceramic coating from severe wear. High-magnification SEM images show that the third-body layer (debris accumulation layer) exhibits spallation, slight grooves, oxide particles, and cracks. This is because, after the third-body layer reaches a certain thickness, parts of it break and detach in the form of fine particles under cyclic shear stress [25]. This indicates that as the wear process proceeds, the dominant wear mode gradually transitions from slight plowing wear to three-body wear and oxidative wear. After SAM/MWCNTs modification (Figure 9c), a third-body layer formed by debris accumulation still exists on the worn surface, and under cyclic loading, stress concentration occurs inside the third-body layer, leading to the initiation and propagation of microcracks and eventually forming visible branched cracks. This further demonstrates that the third-body layer effectively protects the PEO-SAM composite coating. Accordingly, the wear mechanisms under this condition are mainly three-body wear and fatigue wear.

EDS analysis of the two-phase worn areas reveals that no zirconium element (originating from the ZrO_2_ counterface) is detected in the worn areas of the bare composite, indicating that only the substrate has undergone significant wear. After PEO and SAM treatments, different amounts of zirconium are detected in the third-body layer of the worn areas, indicating that both the coating and the counterface have experienced various degrees of wear. Notably, the zirconium content in the worn area of the coating on the Mg side is higher than that on the TC4 side. From the perspective of material hardness, the coating on the TC4 side has a higher hardness and better wear resistance, so its loose surface layer is removed when sliding against ZrO_2_. In contrast, although the coating on the Mg side has a lower hardness, its loose surface layer remains clearly visible under the protection of debris accumulation, and the retained debris promotes the retention of more zirconium in the worn area on the Mg side. In addition, a high oxygen content is detected in the worn areas of both phases, which is attributed to the high local temperature generated by intense friction during sliding wear, causing oxidation of the debris detached from the counterface and the coating surface.

### 3.4. Wear Mechanism Analysis

**Figure 10:**
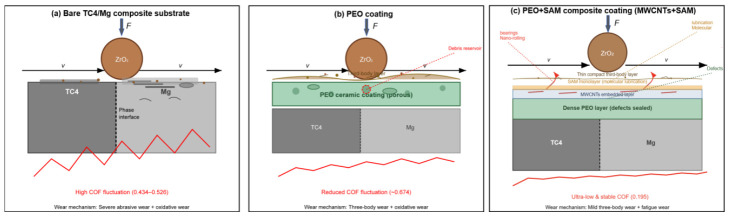
Schematic diagram of wear mechanisms: (**a**) S0; (**b**) S1; (**c**) S2.

Figure 10 schematically illustrates the wear mechanisms and protection mechanisms of the TC4/Mg three-dimensional interpenetrating composite substrate, the PEO coating, and the PEO + SAM composite coating during the ball-on-disk wear test.

As shown in Figure 10a, the wear behavior of the TC4/Mg composite substrate is mainly governed by the hardness difference between the two phases. Under the tangential stress of the friction pair (ZrO_2_ ball), the TC4 phase (hard phase) and the Mg phase (soft phase) exhibit significantly different wear responses: only shallow wear tracks are generated on the TC4 phase surface, while deep and wide grooves and large-area material exfoliation occur on the Mg phase surface. When the friction contact point switches between the two phases and their interface, the discontinuity in friction characteristics leads to substantial fluctuations in the friction coefficient (COF), ranging from 0.434 to 0.526. During the wear process, debris detached from the surfaces of the two phases accumulates at the contact interface, exerting a continuous ploughing effect on the softer Mg phase under tangential force, resulting in typical abrasive wear. Meanwhile, localized oxidation caused by frictional heat further aggravates material loss. Therefore, the dominant wear mechanism of the substrate is severe abrasive wear accompanied by oxidative wear.

After PEO treatment, a porous ceramic layer mainly composed of Mg_2_SiO_4_, MgO, and TiO_2_ is formed on the coating surface (Figure 10b). This coating effectively isolates the substrate from direct contact with the friction pair, significantly reducing the COF fluctuation (average COF of 0.674). Its porous structure plays a key role during the wear process: the debris generated in the initial wear stage is stored in the micropores of the coating. As the friction cycles proceed, this debris is compacted and oxidized under the synergistic action of contact stress and frictional heat, gradually forming a dense third-body layer on the coating surface. This third-body layer buffers the direct cutting of the coating by the friction pair and transforms the wear mode from original abrasive wear to three-body wear and oxidative wear. However, when the third-body layer thickens beyond a critical value, local exfoliation and micro-crack propagation occur under cyclic shear stress, leading to small residual fluctuations in the COF. Overall, the PEO coating effectively reduces the wear rate through a “debris storage–compaction–film formation” mechanism, but its inherent micro-pore defects limit further improvement in wear resistance.

After introducing MWCNT-doped SAM composite modification on the basis of the PEO coating, a fundamental transformation in the wear mechanism is achieved (Figure 10c). First, the nano-filling effect of MWCNTs and the molecular-level coverage of SAM synergistically seal the micropores and cracks of the PEO coating, forming a continuous and dense surface that eliminates stress concentration sources. Second, MWCNTs act as “micro-rolling bearings” at the friction interface: their unique nanotubular structure enables rolling between the coating and the counterpart ball, converting part of the sliding friction into rolling friction and significantly reducing the COF. At the same time, by virtue of their high specific surface area and strong physical adsorption capacity, MWCNTs capture and immobilize the small amount of debris generated during friction, preventing its accumulation at the interface to form a destructive third-body layer. The SAM monolayer, through chemical bonding, forms a low-surface-energy molecular lubrication film on the surface, further reducing adhesion tendency. Under this synergistic effect, the COF decreases to 0.195 and remains stable throughout the test, and the wear volume is reduced by 93.53% compared with the substrate. Only an extremely thin uniform third-body layer and fine network fatigue cracks are observed on the worn surface, with no grooves or exfoliation. Therefore, the dominant wear mechanism of this composite coating is mild three-body wear accompanied by fatigue wear, achieving a transition from a “sacrificial” type to a “precision protection” type.

In summary, from the substrate to the PEO coating and then to the PEO + SAM composite coating, the wear mechanism undergoes a progressive transition from severe abrasive wear to three-body/oxidative wear and then to mild three-body/fatigue wear. The synergistic introduction of MWCNTs and SAM is the key to breaking through the wear resistance bottleneck of conventional PEO coatings, providing a theoretical basis for the application of dual-phase interpenetrating composites in physiological friction environments.

### 3.5. Analysis of Coating Corrosion Resistance

**Figure 11:**
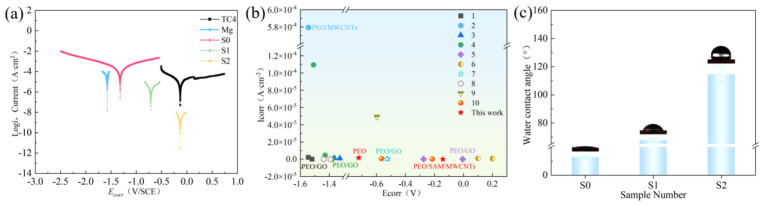
(**a**) ζ−potential polarization curve in 3.5 wt.% NaCl solution, (**b**) comparison of corrosion potentials and current densities for different surface treatment methods: icons 1–10 correspond to references [40,41,42,43,44,45,46,47,48,49], and (**c**) wettability angle.

Figure 11 shows the potentiodynamic polarization curves (Tafel curves) and water contact angles of the TC4/Mg three-dimensional interpenetrating composite and its surface coatings in 3.5 wt.% NaCl solution. The polarization curves (Figure 11a) were fitted using the Tafel extrapolation method to obtain the corrosion potential (Ecorr) and corrosion current density (Icorr), and the results are summarized in Table 1. Generally, a higher corrosion potential indicates that the material is thermodynamically less prone to corrosion, while a lower corrosion current density reflects a slower corrosion reaction rate, i.e., the material has better corrosion resistance.

As shown in Table 1, the Ecorr and Icorr of the TC4 alloy are −0.126 V and 9.29 × 10^−6^ A·cm^−2^, respectively; those of the Mg alloy are −1.57 V and 1.8 × 10^−5^ A·cm^−2^, respectively; and those of the TC4/Mg three-dimensional interpenetrating composite are −1.349 V and 2 × 10^−4^ A·cm^−2^, respectively. It can be seen that the TC4 alloy exhibits the highest corrosion potential, the Mg alloy the lowest, and the composite falls in between. This difference is mainly attributed to the different properties of the surface films on the materials. TC4 can form a dense and stable titanium oxide film. As an n-type semiconductor, the TiO_2_ film can form a high-resistance space charge layer under anodic polarization, hindering electron transfer from the metal substrate to the solution phase. Meanwhile, the dense TiO_2_ film has an extremely low diffusion coefficient for aggressive ions such as Cl^−^, enabling it to maintain long-term stability even in chlorine-containing environments [39]. In contrast, the oxide film on the Mg surface is loose and porous, providing limited protection to the substrate and failing to prevent contact between the corrosive medium and the metal, which results in a low corrosion potential for Mg and easy corrosion occurrence. The composition, structure, and properties of the surface film on the TC4/Mg composite are different from those on TC4 or Mg alone. It contains titanium oxide from TC4 oxidation, products from Mg corrosion, and possibly new substances formed by their interaction. The protective performance of this film lies between that of the TC4 surface film and the Mg surface film, placing the corrosion potential of the composite in an intermediate position. It is worth noting that although the corrosion potential of the TC4/Mg composite is higher than that of Mg, its corrosion current density is one order of magnitude higher than that of Mg. This is because there is a potential difference between Mg and TC4: Mg has a lower potential and acts as the anode, undergoing oxidation and losing electrons, while TC4 has a higher potential and acts as the cathode, promoting a reduction in oxidants in the solution on its surface. The presence of this galvanic couple accelerates electron transfer, making the corrosion reaction more facile and increasing the corrosion current density. After PEO treatment, the Ecorr of the composite increases to −0.722 V, and the Icorr significantly decreases to 1.663 × 10^−6^ A·cm^−2^. After further SAM composite modification, the Ecorr further increases to −0.142 V, and the Icorr decreases to 1.401 × 10^−9^ A·cm^−2^. Compared with the untreated TC4/Mg composite, the Icorr of the PEO + SAM composite coating is reduced by approximately five orders of magnitude (from 2 × 10^−4^ A·cm^−2^ to 1.401 × 10^−9^ A·cm^−2^), corresponding to a protection efficiency of 99.99%. This result indicates that the PEO coating and SAM composite modification significantly enhance the corrosion resistance of the composite, and their synergistic effect effectively seals the micropores and defects in the coating, blocking the penetration path of the corrosive medium. Figure 11b further compares the corrosion resistance achieved by different processes. The PEO and PEO/SAM/MWCNTs composite coatings prepared on the surface of the TC4/Mg three-dimensional interpenetrating material (which is susceptible to galvanic corrosion) exhibit corrosion resistance comparable to, and in some cases even superior to, that of PEO composite coatings on single-phase light alloy substrates [40,41,42,43,44,45,46,47,48,49].

Figure 11c shows the water contact angle (WCA) measurement results on the surfaces of the different samples. The untreated TC4/Mg composite surface exhibits hydrophilicity, with a contact angle of only 12.6 ± 0.5°, which is attributed to the presence of many polar groups and a relatively high roughness on the pristine surface, making it easily wettable by water. After PEO treatment, the surface contact angle increases to 67.7 ± 1.4°; the surface remains hydrophilic but with significantly reduced wettability. This is ascribed to the inherent hydrophilicity of the micro-/nano-porous structure and the ceramic components (MgO, TiO_2_, etc.) of the PEO coating [50], while the micro-/nano-roughness suppresses droplet spreading to some extent. After further introduction of the SAM self-assembled monolayer (doped with MWCNTs), the contact angle dramatically rises to 114.6 ± 4.9°, achieving a transition from hydrophilicity to hydrophobicity. This remarkable improvement in wettability mainly originates from two synergistic effects. On the one hand, SAM molecules (long-chain silanes) form a dense and well-ordered hydrophobic molecular layer on the PEO coating surface via chemical bonding, and their low surface energy reduces the solid–liquid interaction [51,52]. On the other hand, the filling effect of MWCNTs retains the original micro-/nano-rough structure of the PEO coating; together with the low-surface-energy chemical modification, a micro-/nano-composite structure similar to the “lotus leaf” effect is built on the surface, causing water droplets to adopt the Cassie-Baxter state [53] and thus yielding a high contact angle. The significant increase in contact angle implies that the corrosive medium (aqueous Cl^−^-containing solution) is more difficult to spread and penetrate on the coating surface, which further blocks the contact path between the electrolyte and the substrate. This is an important complementary mechanism for the greatly enhanced corrosion resistance (five-order-of-magnitude reduction in Icorr) of the SAM/MWCNTs composite coating.

**Figure 12:**
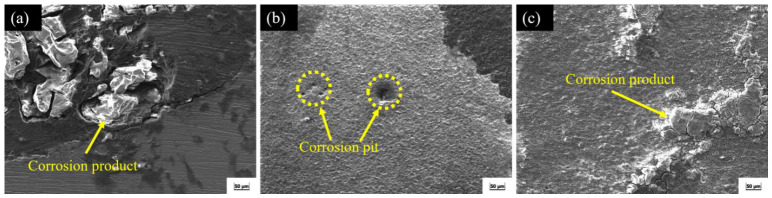
Microcorrosion morphology after dynamic potential polarization test; (**a**) S0; (**b**) S1; (**c**) S2.

Figure 12 shows the surface morphologies of the TC4/Mg composite and its surface coatings after corrosion testing. For the untreated TC4/Mg composite, extensive pitting, interface cracking, and exfoliation of corrosion products are observed on the substrate surface. Corrosion rapidly propagates along the two-phase interface, forming obvious localized corrosion channels. After PEO treatment, the coating surface is largely uniform and intact, with only a few individual corrosion pits. After further PEO + SAM composite modification, accumulation of corrosion products is observed on the surface, but no obvious pitting, cracks, or signs of medium erosion are visible, and the surface remains intact overall.

**Figure 13:**
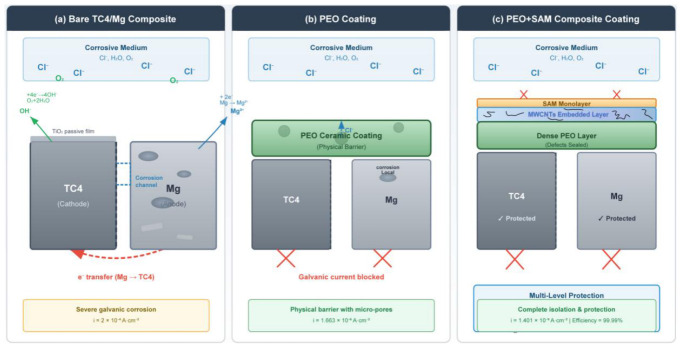
Schematic diagram of corrosion; (**a**) S0; (**b**) S1; (**c**) S2.

Figure 13 shows a schematic diagram of the corrosion mechanism. In the TC4/Mg composite, there is a significant potential difference between the titanium alloy reinforcement phase and the magnesium matrix, which readily forms a strong galvanic corrosion system in a corrosive medium. The magnesium matrix acts as the anode and continuously dissolves (Mg → Mg^2+^ + 2e^−^), while TC4 serves as the cathode and promotes the oxygen reduction reaction (O_2_ + 2H_2_O + 4e^−^ → 4OH^−^). A stable galvanic couple is formed between the two phases, significantly accelerating the corrosion failure process of the matrix. At the same time, the natural oxide film is loose and discontinuous, unable to effectively block the ingress of aggressive ions, further exacerbating the initiation and development of corrosion. After PEO treatment, a ceramic coating composed mainly of TiO_2_, MgO, and other oxides is formed in situ on the surface of the composite. This coating is dense and has high bonding strength, acting as a stable physical barrier that effectively isolates the matrix from the corrosive medium and significantly weakens or even blocks the galvanic coupling between TC4 and Mg, thereby improving the corrosion resistance. After further modification with a self-assembled monolayer (SAM) doped with multi-walled carbon nanotubes (MWCNTs) on the PEO coating, the MWCNTs, by virtue of their high aspect ratio and nano-sized filling effect, precisely seal the residual micropores and micro-defects in the PEO coating, cutting off the permeation paths of corrosive ions. At the same time, the SAM molecules form a dense and well-ordered molecular barrier on the coating surface through chemical bonding, enhancing the interfacial adhesion and hindering the electron transfer process required for the corrosion reaction. In addition, the SAM layer endows the coating surface with high hydrophobicity (contact angle of 114.6°), making it difficult for the water-based corrosive medium to spread and stay on the surface, thereby further inhibiting corrosion from the perspective of dynamic wetting. Under the synergistic effect, the corrosion potential of the system shifts significantly toward the positive direction, the corrosion current density decreases, and the corrosion rate is suppressed to an extremely low level. This composite treatment constructs a multi-level synergistic protection system of “ceramic barrier layer + nano-sealing + molecular protection”, which jointly inhibits corrosion from multiple aspects including physical isolation, defect sealing, interface stabilization, and electron transfer blocking, achieving efficient and long-term protection of the TC4/Mg composite.

## 4. Conclusions

(1) PEO coating: A ceramic layer mainly composed of Mg_2_SiO_4_, MgO, and TiO_2_ is formed in situ on the surface of the TC4/Mg composite, transforming the open porous structure into a physical barrier that effectively isolates the corrosive medium and weakens the TC4-Mg galvanic coupling. Meanwhile, its porous structure can store wear debris during friction, which is compacted and oxidized to form a third-body protective layer, reducing the wear volume by 89%.
(2) SAM composite coating (MWCNTs + SAM): MWCNTs seal the micropore defects of the PEO coating through a nano-filling effect and act as “micro-rolling bearings” as well as debris adsorbents during friction, reducing the friction coefficient to 0.195 (82.35% lower than that of the substrate). The SAM monolayer forms a dense molecular barrier via chemical bonding, further hindering electron transfer and ion penetration.
(3) Synergistic effect: PEO provides the bottom barrier and debris-retaining framework, while MWCNTs and SAM achieve a synergistic combination of defect sealing and molecular blocking, forming a stepwise protection from micrometer to molecular scales. The corrosion current density decreases from 2 × 10^−4^ A (for the bare composite) to 1.401 × 10^−9^ A (a reduction by five orders of magnitude), with a protection efficiency of 99.99%. The wear volume is reduced by 93.53% compared with the bare composite.

## Figures and Tables

**Table 1:** Corrosion Potential and Corrosion Current Density.

Material	Ecorr (V)	Icorr (A·cm^−2^)
TC4	−0.126	9.29 × 10^−6^
Mg	−1.57	1.8 × 10^−5^
S0	−1.349	2 × 10^−4^
S1	−0.722	1.663 × 10^−6^
S2	−0.142	1.401 × 10^−9^

## Data Availability

The original contributions presented in this study are included in the article. Further inquiries can be directed to the corresponding authors.

## Abbreviations

The following abbreviations are used in this manuscript:

TC4	Ti-6Al-4V titanium alloy
Mg	Magnesium
PEO	Plasma electrolytic oxidation
SAM	Self-assembled monolayer
MWCNTs	Multi-walled carbon nanotubes
COF	Coefficient of friction
SEM	Scanning electron microscopy
EDS	Energy-dispersive X-ray spectroscopy
XRD	X-ray diffraction
MMIPCs	Metal–metal interpenetrating phase composites
AZ31	AZ31 magnesium alloy
DPTMS	(3-(2,3-Epoxypropoxy)propyl)trimethoxysilane
EDTA-2Na	Ethylenediaminetetraacetic acid disodium salt
NaCl	Sodium chloride
ZrO_2_	Zirconium dioxide
